# Where three snail species attach while emersed in relation to heterogenous substrate temperatures underneath intertidal boulders

**DOI:** 10.7717/peerj.11675

**Published:** 2021-07-09

**Authors:** Nathan Janetzki, Kirsten Benkendorff, Peter G. Fairweather

**Affiliations:** 1 College of Science and Engineering, Flinders University, Adelaide, SA, Australia; 2 National Marine Science Centre, Faculty of Science and Engineering, Southern Cross University, Coffs Harbour, NSW, Australia

**Keywords:** Gastropod, Thermal ecology, Gradient, Mosaic, Rock characteristics, Thermal imagery

## Abstract

Mobile intertidal gastropods can employ behavioural thermoregulation to mitigate thermal stress, which may include retreating under boulders when emersed. However, little is known about how gastropod occupancy of under-boulder habitats is associated with any variations in substrate temperature that exist under boulders. Thermal imagery was used to measure the temperature of boulder lower surfaces and investigate how three snail species were associated at low tide with the maximum and average temperatures underneath grey siltstone and quartzite. Lower boulder surfaces had heterogeneous temperatures, with grey siltstone having temperature gradients and quartzite temperature showing mosaics. Temperature differences between the hottest and coolest gradient or mosaic locations were >5 °C; thus there was a range of temperatures that snails could interact with. All three snail species occupied cooler parts of temperature mosaics or gradients, avoiding the hottest areas. Stronger associations were detected on the hotter grey siltstone and for the more-thermally sensitive *Nerita atramentosa* and *Diloma concameratum*. Even though snails were associated with cooler areas, some individuals were still exposed to extreme substratum heat (>50 °C). These results suggest that gastropod thermoregulatory behaviour is far more complex than simply retreating underneath boulders at low tide, as there is also a range of under-boulder temperatures that they interact with. Untangling interactions between intertidal gastropods and heterogenous substrate temperatures is important given rocky seashores already represent a thermally-variable and potentially-stressful habitat, which may be exacerbated further given predictions of warming temperatures associated with climate change.

## Introduction

Intertidal gastropods are periodically exposed to the terrestrial environment during low tide and, as marine ectotherms, do not physiologically regulate their body temperature; instead their body temperature tracks that of the surrounding environment (Wolcott, 1973; Madeira et al., 2012). Many intertidal gastropods live in close contact with the substratum, with their large muscular foot keeping them thermally coupled to the substratum underneath (Vermeij, 1971; McMahon, 1990; Miller et al., 2015). Consequently, gastropod body temperature is often positively correlated with substratum temperature at low tide (Soto & Bozinovic, 1998; Caddy-Retalic, Benkendorff & Fairweather, 2011; Chapperon & Seuront, 2011a). Rock surfaces exposed to direct insolation when emersed can warm by 10–20 °C (Southward, 1958; Harris, 1990; Bertness, 1999; Helmuth & Hofmann, 2001), with gastropods inhabiting the mid and upper levels of the seashore spending 14–98% of their lives in these thermally-challenging conditions (McMahon, 1988; Harley, 2003; Helmuth et al., 2006b). Thus, rocky seashores represent a thermally-variable and potentially-stressful habitat for the gastropod populations inhabiting them (Helmuth & Hofmann, 2001; Harley, 2008).

For intertidal gastropods, exposure to extreme heat and low humidity when emersed can challenge their development, survival and ultimately persistence through heat stress, desiccation, or a combination of both (Bertness, 1999; Helmuth & Hofmann, 2001; Harley, 2003). Biotic responses to extreme heat are highly variable, often differing among species, conspecifics, populations, or life stages of the same species (Pörtner, 2001; Przeslawski, 2004; Byrne & Przeslawski, 2013; Dong et al., 2017). Depending on the intensity, rate of warming, and duration of extreme heat events (Evans, 1948; Pörtner, 2001; Moyen, Somero & Denny, 2019), both non-lethal and lethal impacts may result from exposure to sub-optimal heat. Non-lethal impacts can include reduced growth rates (Jones & Boulding, 1999; Lathlean, Ayre & Minchinton, 2013; Lamb, Leslie & Shinen, 2014) or the onset of heat coma (Evans, 1948; McMahon, 1990; Miller et al., 2015); while lethal impacts can include mass mortality events during heatwaves (Harley, 2008; Helmuth et al., 2002; Seuront et al., 2019), overall lower survival rates in less thermally-favourable habitats (Jones & Boulding, 1999; Harley, 2008; Gedan et al., 2011; Leal et al., 2020), or limitations on the vertical seashore distribution of some species (Wolcott, 1973; Raimondi, 1988; Somero, 2002; Harley, 2003).

Due to their physiological limitations with regulating body temperature, intertidal gastropods can instead employ behavioural thermoregulation to help mitigate some of the deleterious impacts associated with exposure to extreme heat during emersion (Soto & Bozinovic, 1998; Ng et al., 2017; Hayford, O’Donnell & Carrington, 2018). Each thermoregulatory behaviour employed by intertidal ectotherms includes either a fight-or-flight response sensu Ng et al. (2017). A fight response, where organisms can modify their own environment to minimise thermal extremes (Ng et al., 2017), may include: shell-posturing behaviour (Seuront & Ng, 2016; Chapperon, Studerus & Clavier, 2017; Ng et al., 2017); aggregating with conspecifics (Underwood, 1976; Chapperon, Le Bris & Seuront, 2013); remaining inactive at low tide by withdrawing into the shell and cementing the aperture to the substratum with mucus (Vermeij, 1971; McMahon, 1990); or attaching to the substratum with a mucus holdfast (Marshall & Chua, 2012; Seuront, Ng & Lathlean, 2018). A flight response, where organisms limit exposure to extreme heat by retreating to cooler habitats (Ng et al., 2017), may include taking refuge: on the lower surfaces of boulders or mangrove roots (Chapperon & Seuront, 2011a, 2011b); in pits or crevices on rock platforms (Garrity, 1984; Bates & Hicks, 2005); on substrate surfaces orientated away (i.e. shaded) from direct insolation (Williams & Morritt, 1995); or in rock pools, algal or barnacle patches (Evans, 1948; Jones & Boulding, 1999).

Small boulders (maximum dimension ≤ 30 cm) represent one type of habitat that gastropods may seek refuge under during low tide. Janetzki, Benkendorff & Fairweather (2021) found that substrate temperature can be highly variable underneath boulders, with differences of up to 15.5 °C between the hottest and coolest areas on a lower boulder surface. Moreover, the thermal quality of under-boulder habitats was influenced by lithology, with rock types differing in both maximum temperature and the qualitative patterns of temperature that developed across boulder surfaces when emersed (Janetzki, Benkendorff & Fairweather, 2021). Consequently, gastropod thermoregulatory behaviour may extend beyond simply retreating under boulders, as organisms may also respond to the heterogenous temperatures underneath them. However, it is currently unclear whether the locations occupied by gastropods underneath boulders during low tide are in any way associated with these heterogenous surface temperatures. Some evidence for biota responding to small-scale temperature differences has been identified elsewhere, with Soto & Bozinovic (1998) reporting that periwinkles avoided the hottest areas of substrate when offered a centimetre-scale temperature gradient of warmer through cooler locations under laboratory conditions. Moreover, in the terrestrial realm Huey et al. (1989) found that garter snakes adjusted their positioning under rocks (<2 m length or width) in response to heterogenous temperatures.

To determine whether gastropod occupancy under boulders is associated with the patterns of heterogenous surface temperature identified by Janetzki, Benkendorff & Fairweather (2021), a field study of their experimental results was completed. We first established whether the qualitative patterns of surface temperature (definitions and representative images of these qualitative patterns are supplied in the methods) and rock-type differences in maximum surface temperature identified under experimental conditions (Janetzki, Benkendorff & Fairweather, 2021), also occur for boulders on the seashore. We then investigated how the specific locations occupied by three snail species underneath boulders were related to any patterns of heterogenous surface temperature. As in previous studies (see Monaco et al., 2015), we focused primarily on maxima due to extreme temperatures having a greater impact on organism survival and fitness (e.g. Jones & Boulding, 1999; Harley, 2008; Gedan et al., 2011). Boulders of two rock types were sampled because surface temperatures can differ among rock types (Janetzki, Benkendorff & Fairweather, 2021), and this in turn can influence ectotherm responses to substratum temperature (Raimondi, 1988; Marshall, McQuaid & Williams, 2010; Judge, Botton & Hamilton, 2011). Untangling associations between intertidal gastropods and substratum temperatures is important given predictions of hotter air temperatures and an increased frequency of extreme heat events associated with global climate change (IPCC, 2013). Such changes in climate may exacerbate the temperature extremes and variability currently experienced by intertidal ectotherms while emersed, which may be problematic given some intertidal biota already live near or at their upper tolerable thermal limits (Somero, 2002; Helmuth et al., 2002; Madeira et al., 2012). We tested the following four null hypotheses (H1–H4) to improve our understanding of any low-tide associations between snails and small-scale temperature heterogeneity on boulder lower surfaces:

(H1) emersed boulder lower surfaces will not display patterns of temperature difference;
(H2) temperature characteristics will not differ between grey siltstone and quartzite;
(H3) target temperature (i.e. the substrate temperature where the snail is attached) does not differ from the maximum or average boulder temperature; and
(H4) differences between target temperature and either thermal maxima or minima on boulders do not differ between grey siltstone and quartzite.

## Materials & Methods

### Sampling location

Two intertidal boulderfields along South Australia’s Gulf St Vincent were sampled, grey siltstone at Marino Rocks (35°02′ S, 138°30′E) and quartzite at O’Sullivan Beach (35°07′ S, 138°28′E). Both seashores have a westerly aspect and are located just 20 km apart on the south-eastern side of Gulf St Vincent, a large inverse estuary with a geographic setting that protects the coastline from swell. While both seashores have similar oceanic and environmental conditions such as low wave exposure, a relatively flat shore slope, and are free from shading by cliffs, localised variations in wave splash and wind speed/direction may occur. Janetzki, Benkendorff & Fairweather (2021) showed that grey siltstone was consistently one of the hottest rock types sampled from this region, while quartzite was one of the coolest (Appendix Fig. A1). Hence, these two rock types were selected as they represented the upper and lower extremes of surface temperature variability among seashore rock types for this region.

### Sampling design

Janetzki, Benkendorff & Fairweather (2021) identified three qualitative patterns of temperature difference: mosaics, gradients and limited heterogeneity (Appendix Fig. A2). Limited temperature heterogeneity consisted of only small temperature differences <5 °C between the warmest and coolest areas on a boulder surface (Janetzki, Benkendorff & Fairweather, 2021, Appendix Fig. A2a). Temperature gradients consisted of a gradual decrease in temperature from the boulder side nearest the sun to the side opposite, with a temperature difference between the warmest and coolest gradient areas ≥5 °C (Janetzki, Benkendorff & Fairweather, 2021, Appendix Fig. A2b). Temperature mosaics consisted of heterogeneous temperatures across the entire boulder surface, with a temperature difference between the warmest and coolest mosaic areas ≥5 °C (Janetzki, Benkendorff & Fairweather, 2021, Appendix Fig. A2c).

Two observations regarding the development of temperature gradients and mosaics on boulder surfaces were noted during the common-garden experiment: (1) boulders must be exposed to sunlight to develop temperature differences ≥5 °C; and (2) even small-sized boulders (maximum dimension ≤ 30 cm) still take at least 4 hours to approach their thermal maxima when exposed to uninterrupted sunshine (Janetzki, Benkendorff & Fairweather, 2021; Appendix Fig. A1). Consequently, only sunny days with little to no cloud cover were sampled during this study. Boulders were sampled approximately one hour either side of predicted daytime low tides (low tide ≤ 0.40 m Australian Height Datum), as pilot studies showed boulders had been emersed for at least four hours by this time. Uninterrupted sunshine (i.e. no cloud cover) and daytime low tides are typical for the Gulf St Vincent during summer.

All sampling was completed between December 2016 and February 2017 during the austral summer to target days of extreme aerial and substratum temperatures. Air temperature measurements (1955–2017 inclusive) from the weather station nearest the sampled seashores (Adelaide Airport) showed this region of the coast experiences a mean summer maximum air temperature of approximately 28 °C (Commonwealth of Australia BoM, 2017). Therefore, sampling was completed on four days at each seashore that had maximum air temperatures at the time of low tide above this summer average (i.e. hotter days), and on four days that had maximum air temperatures at or below this summer average (i.e. cooler days) (Table 1). Field studies were approved by Primary Industries & Regions South Australia (PIRSA) under S115 Ministerial Exemption Number 9902638.

**Table 1:** Mean difference (°C) for target—maximum temperature across replicate boulders on each day for *Bembicium nanum, Diloma concameratum* and *Nerita atramentosa* on grey siltstone and quartzite.

Species				*Bembicium nanum*		*Diloma concameratum*		*Nerita atramentosa*	
Rock type	Date	Predicted time of low tide (24 h clock)	Max air temp (°C)	*n*	Target—maximum	*n*	Target—maximum	*n*	Target—maximum
Grey siltstone	20/12/16	14:27	23	30	−4.09	30	−5.61	30	−5.58
	16/12/16	13:07	26	30	−4.89	22	−7.34	30	−7.19
	27/1/17	12:12	27	30	−5.10	30	−6.06	30	−6.23
	10/1/17	11:19	28	28	−4.26	30	−4.96	30	−4.88
	28/1/17	12:32	29	30	−5.66	30	−6.46	30	−6.83
	4/1/17	14:42	33	30	−5.06	19	−6.11	30	−6.98
	5/1/17	15:11	35	30	−4.84	28	−7.22	30	−6.87
	17/1/17	13:52	39	30	−4.67	18	−8.01	30	−7.91
Grand mean					−4.82		−6.47		−6.56
						
Quartzite	18/12/16	13:47	25	30	−4.65	30	−5.13	30	−5.44
	21/12/16	14:56	25	30	−4.36	30	−5.02	30	−4.96
	12/1/17	12:22	28	30	−3.96	30	−5.09	30	−5.26
	3/1/17	14:17	28	30	−4.64	30	−5.40	30	−4.91
	29/1/17	12:52	30	30	−4.68	30	−5.17	30	−5.12
	16/1/17	13:35	34	30	−4.84	24	−5.71	30	−5.20
	9/2/17	12:03	38	30	−4.41	27	−5.13	30	−5.89
	6/1/17	15:46	40	30	−4.29	23	−5.60	30	−5.52
Grand mean					−4.48		−5.28		−5.29

**Note:**

Entries are ordered by the maximum air temperature within each rock type. All mean temperature differences (α = 0.05), as determined by paired *t*-tests, are significant. *n* = the number of snails sampled daily for each species.

Pilot studies found the grazing snails *Nerita atramentosa* (Reeve, 1855), *Diloma concameratum* (W. Wood, 1828), and *Bembicium nanum* (Lamarck, 1822) to be most abundant, with sampling subsequently targeting these three species. McMahon (1990) showed *D. concameratum* had the lowest heat tolerance of these species, reporting a heat coma temperature (HCT) of 35.6 °C in heated seawater. A HCT of 38.9 °C was identified for *N. atramentosa* in heated seawater (McMahon, 1990). *Bembicium nanum* likely has the highest heat tolerance of these three species, although no HCT was identified in the published literature. However, experiments testing the heat tolerance of a congener, *B. vittatum*, identified a HCT of 41.0 °C in heated seawater (McMahon, 1990). Given *B. nanum* is also a higher-shore littorinid (Underwood, 1975), and occurs in similar sub-tropical and temperate habitats as its congener *B. vittatum*, it is likely to have a similar heat tolerance.

**Figure 1:**
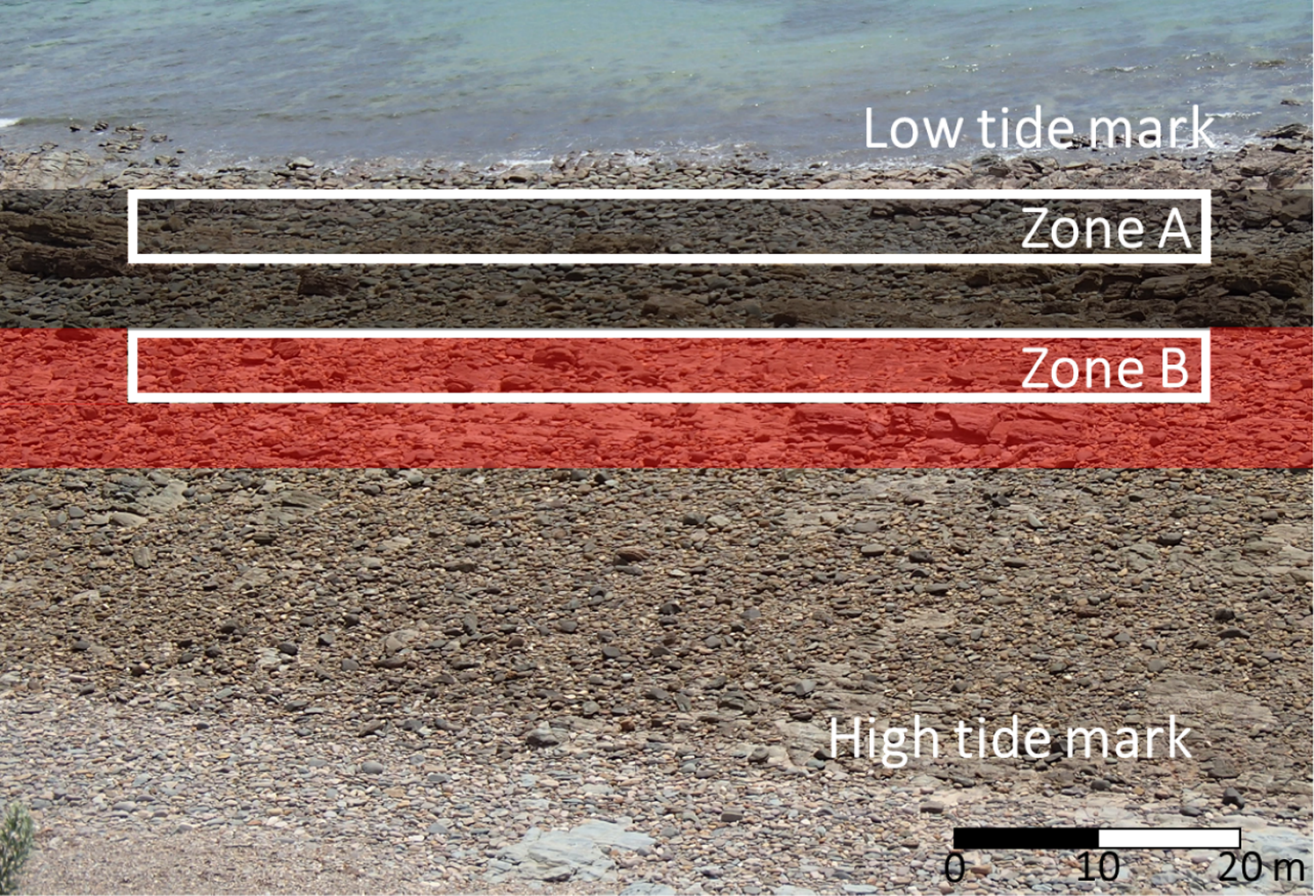
Boulderfield at Marino Rocks. This photograph shows the lower-shore distribution of *Nerita atramentosa* and *Diloma concameratum* (black shaded area) and higher-shore distribution of *Bembicium nanum* (red shaded area), and how two shore-parallel zones (Zones A & B) were used to sample these species-specific seashore distributions.

*Nerita atramentosa* and *D. concameratum* generally had a mean shell length ≥15 mm, and were hence identified as adults, with *N. atramentosa* reaching reproductive maturity at a mean shell length of 13.5 mm (Underwood, 1975). The highest abundances of *N. atramentosa* and *D. concameratum* were recorded lower on the seashore, approximately 5–15 m shoreward of the low-tide mark (Fig. 1). In contrast, *B. nanum* generally had a mean shell breadth ≤5 mm, with individuals identified as juveniles, with this species reaching reproductive maturity at a mean shell breadth of 11 mm (Underwood, 1975). The highest abundances of *B. nanum* occurred higher up the seashore, approximately 15–25 m shoreward of the low-tide mark (Fig. 1). These species-specific seashore distributions mandated that sampling be completed in two shore-parallel zones, with each zone having an across-shore width of 5 m and an along-shore length of 80 m (Fig. 1). One zone, Zone A, was established approximately 5 m shoreward of the low-tide mark to sample adult *N. atramentosa* and *D. concameratum*, while Zone B was established 15 m shoreward of the low tide mark to sample juvenile *B. nanum* (Fig. 1).

Randomly-selected boulders were only sampled if their lower surfaces were inhabited by an individual from one or more target species, with each sampled individual having to occur as an independent organism. In this study, an independent organism was defined as any individual that had no direct physical contact with conspecifics. Consequently, organisms clustered in groups (predominantly *N. atramentosa* and *D. concameratum*) were only sampled if the individual organisms constituting each cluster were not in physical contact with one another, so as to constrain their choice. We chose not investigate any associations between gastropod aggregation and substrate temperature, as these have been investigated previously (e.g. Chapperon, Le Bris & Seuront, 2013; Chapperon, Studerus & Clavier, 2017). Only one individual, from each target species, per lower surface was sampled, with sampled individuals being randomly selected. Multiple target species per surface were sampled if surfaces were cohabited.

To sample lower surfaces, boulders were flipped upside down and shaded from direct sunlight. Boulders were only sampled if snails remained attached during the flipping process. Where possible, 30 under-boulder surfaces inhabited by each target species were sampled at each seashore on each day (Table 1). Target snail species exhibited a patchy and over-dispersed distribution (Chapman & Underwood, 1996; Chapman, 2005), with many surfaces unoccupied by any target species. Zone A was always sampled before Zone B to ensure all boulders were sampled before they were submersed by the incoming tide (Fig. 1).

**Figure 2:**
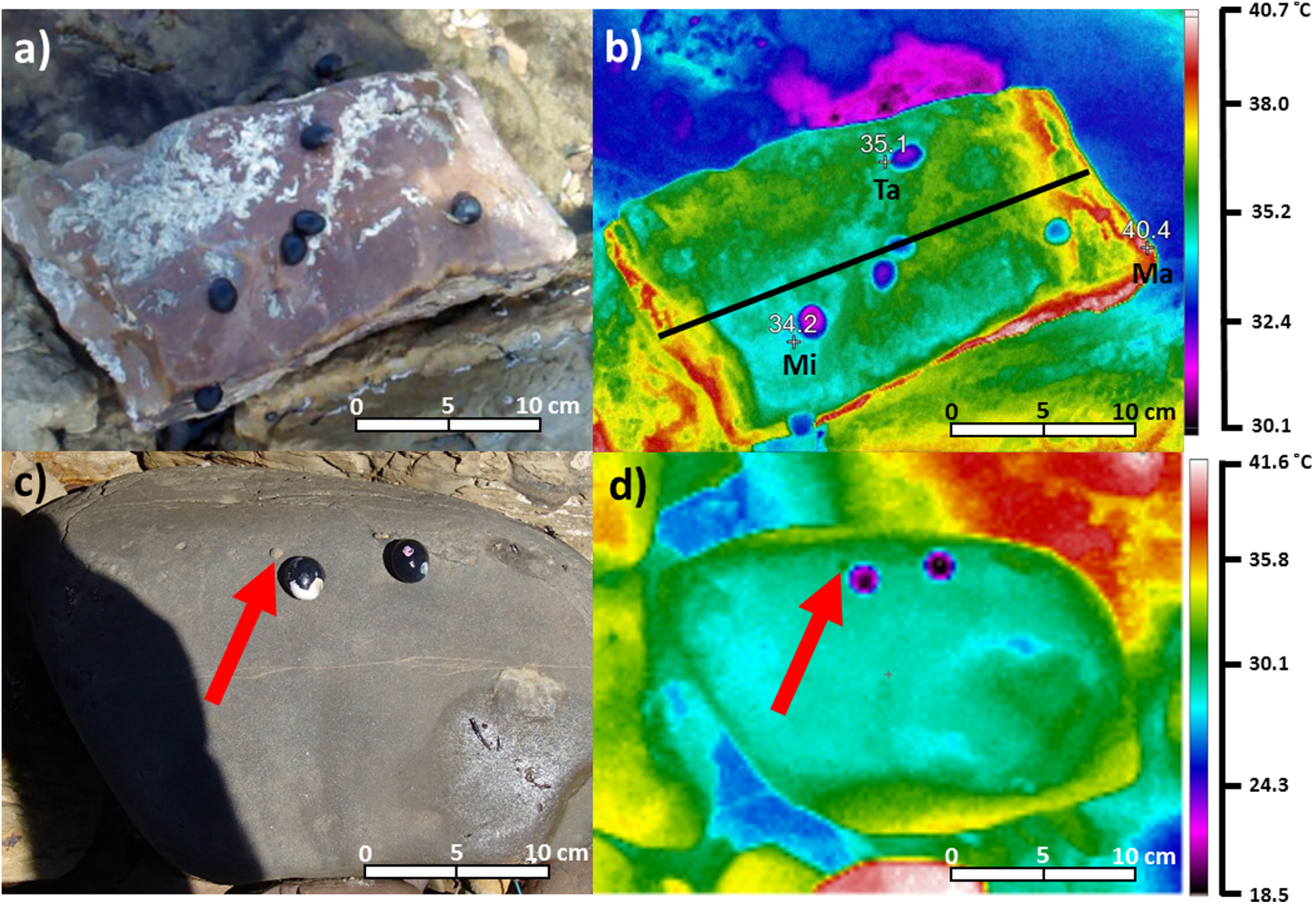
Digital photograph (A) and thermal image (B) of *Nerita atramentosa* on the lower surface of a quartzite boulder (range: 40.4–34.2 = 6.2 °C). Ma = maximum surface temperature, Mi = minimum surface temperature and Ta = target temperature for an individual *N. atramentosa*. The solid black line in (B) denotes how transects were drawn on each image to quantify patterns of temperature difference. Digital photograph (C) and thermal image (D) showing the use of two chilled *N. atramentosa* shells to assist with locating a juvenile *Bembicium nanum* (one marked with the red arrow) on the lower surface of a siltstone boulder (range: 36.9–25.8 = 11.1 °C).

Imagery was used to capture associations between snails and substrate temperatures in situ. For every sampled surface (*N* = 1152), a digital photograph and a thermal image were recorded, and archived images were subsequently processed in the laboratory. Digital photographs were captured using an Olympus Tough TG-820 digital camera, and were used as a reference to identify individual snails (Fig. 2). Thermal images were captured using a Ti20 Fluke thermal imaging camera, using the same camera settings described by Janetzki, Benkendorff & Fairweather (2021). The thermal resolution of this camera was ≤0.2 °C at 30 °C, with an accuracy to 2% or 2 °C, whichever was greater (Janetzki, Benkendorff & Fairweather, 2021). Default camera settings were employed, including emissivity, which was set at 0.95 (Janetzki, Benkendorff & Fairweather, 2021). As detailed in Janetzki, Benkendorff & Fairweather (2021), this default emissivity was applied, even though emissivity may have differed within and among the rock types sampled, as previous thermography studies in the long-infrared range (9–14 µm) have shown that the emissivity of dry rock generally ranges between 0.95 and 1 (Rivard, Thomas & Giroux, 1995; Danov, Vitchko & Stoyanov, 2007; Cox & Smith, 2011; Lathlean, Ayre & Minchinton, 2012). To avoid measuring inaccurate temperatures from increased amounts of thermal energy being reflected by wet surfaces (Lathlean, Ayre & Minchinton, 2012; Lathlean & Seuront, 2014; Seuront, Ng & Lathlean, 2018), boulders from wet habitats (i.e. rock pools or wet sediment) were not sampled. As boulders had been emersed for at least 4 h before being sampled, and previous research has confirmed these types of boulders take no longer than 5–10 min to dry once emersed as water rarely permeated their surfaces (Janetzki, Benkendorff & Fairweather, 2021), differential rates of drying among individual boulders or rock types was not a confounding variable.

Due to their large size and shell temperatures generally somewhat cooler than the substrate, *N. atramentosa* and *D. concameratum* were easy to locate in archived thermal images (Fig. 2). In contrast, the smaller *B. nanum* generally had shell temperatures more similar to the substrate, and were often difficult to locate on the image (Fig. 2). To rectify this, empty *N. atramentosa* shells were chilled on ice and were then placed approximately 1 cm to the lower right of *B. nanum* individuals when recording thermal images (Fig. 2). As the chilled shells were much cooler than the substrate, they acted as an easily-identifiable reference point, making it easier to locate *B. nanum* in archived thermal images (Fig. 2).

Archived thermal images were processed using the InsideIR version 4.0 software (Fluke Corporation, Everett, WA, USA). For each image of lower surfaces, maximum, minimum and average boulder temperatures were quantified by tracing around each boulder. A temperature range (i.e. maximum − minimum = range) for each surface was then quantified (daily *n* = 62–80, Fig. 2). A target temperature was recorded for each sampled snail, and, for purposes of standardisation, was measured to the immediate left of each snail (Fig. 2). The maximum, minimum, and average temperatures of each lower surface were subtracted from each target temperature, to determine how snails were associated with substratum temperatures. Due to the low probability that individual snails will be randomly recorded only in areas with the maximum (or minimum) substratum temperature, comparisons between snail target temperatures and average substrate temperatures were also completed to better test the hypothesis that snails occupy cooler areas on boulder surfaces.

Within each traced boulder perimeter, a transect was drawn from the centre of the boulder side facing the sun to the centre of the side opposite (Fig. 2). This was done to verify whether the three qualitative patterns of temperature heterogeneity (gradients, mosaics, limited heterogeneity) described by Janetzki, Benkendorff & Fairweather (2021) also occur for boulders on the seashore. Transects were drawn for 20 randomly-selected images for three days per seashore, spanning the range of maximum daily air temperatures sampled. This subset of boulders was deemed sufficient to quantify the frequency of occurrence for these temperature patterns, as Janetzki, Benkendorff & Fairweather (2021) showed that their occurrence was primarily influenced by the presence/absence of cloud cover, which was not a factor here as only sunny days were sampled.

### Statistical analyses

Analyses were completed using either PRIMER v7/PERMANOVA+ (PRIMER-e, Plymouth, UK) or SYSTAT v13 (Systat Software Inc) statistical software, with significance set at α = 0.05, unless otherwise noted. To establish whether boulders on the seashore developed heterogenous temperatures (H1), frequencies of occurrence (%) for each temperature pattern (gradients, mosaics, limited heterogeneity) were tallied separately for 20 boulders per seashore, on each day sampled. Temperature pattern tallies were then pooled across the three days sampled per rock type, with a Chi-square two-way contingency table analysis testing (α = 0.05) for any associations between rock type and temperature patterns using these pooled tallies.

To test whether temperature characteristics differed between rock types (H2), means for boulder temperature range and maxima on each sampling day were used in separate univariate one-factor PERMutational ANalyses of COVAriance (PERMANCOVA). Analyses were completed using their untransformed means, with Euclidean distance resemblance matrices prepared separately for each temperature characteristic as dependent variables. To test whether mean temperature characteristics were related to air temperature, maximum daily air temperatures were used as the co-variate in PERMANCOVA models. Permutations of residuals were completed using a reduced model with 9,999 permutations.

To test whether snail target temperatures were significantly different from the maximum or average temperature of boulders (H3), paired *t*-tests were completed for each target species on each day sampled for each rock type. As the same target temperatures were used in comparisons against maximum and average temperatures, a Bonferroni correction was applied to reduce the likelihood of making a Type I error. Consequently, a corrected significance level of α = 0.025 was applied. Means were then calculated from the replicate boulders on each day for the difference between target temperatures and boulder maxima, and between target temperatures and boulder minima. Mean differences were also used in one-factor PERMANCOVA, as described above (with air temperature as the covariate), to test for differences between grey siltstone and quartzite in how each snail species was associated with the temperature range on lower surfaces (H4). As the same mean target temperature was used to calculate each temperature difference measure for each species, a Bonferroni correction (corrected α = 0.025) was applied.

## Results

### Emersed boulder lower surfaces have a heterogenous surface temperature

**Figure 3:**
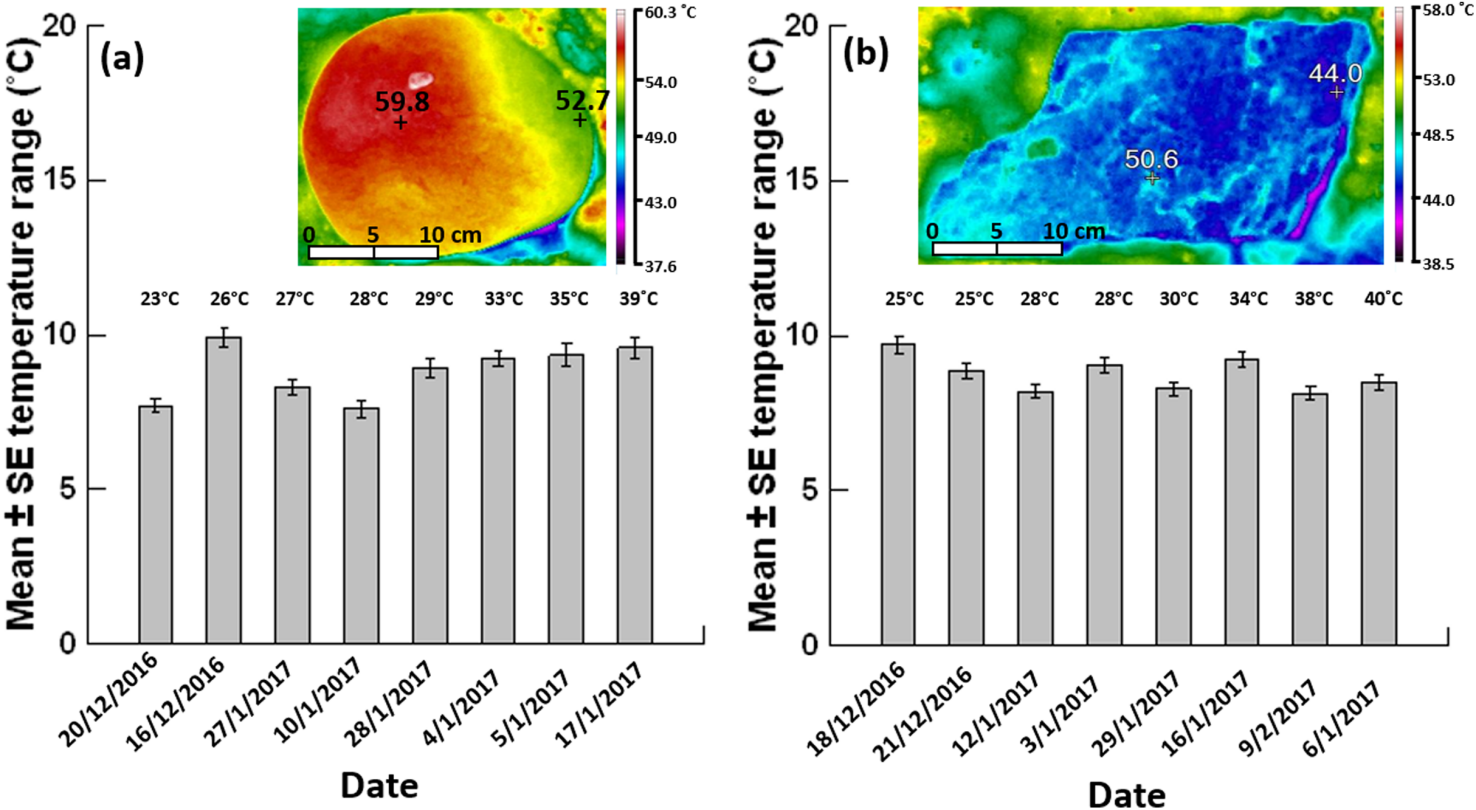
Mean ± SE temperature range (n = 62–80 boulders per day). (A) Grey siltstone and (B) quartzite on different days (ordered by the daily maximum air temperature during sampling at low tide, specified above each individual bar) during the 2016–2017 austral summer. Inset thermal images above each bar chart show a representative temperature gradient on a siltstone boulder (range: 59.8–52.7 = 7.1 °C) and a temperature mosaic on a quartzite boulder (range: 50.6–44.0 = 6.6 °C) exposed to 4 h sunshine at an air temperature of 39 °C.

The lower surfaces of grey siltstone and quartzite boulders generally had a heterogeneous surface temperature. The mean range of this heterogeneous temperature differed by >5 °C on each day sampled at each seashore (Fig. 3). The type of qualitative temperature pattern identified on lower surfaces differed between rock types (Pearson Chi-square *p*-value = <0.001), with siltstone generally developing gradients (Fig. 3A) whereas quartzite developed mosaics (Fig. 3B). Quartzite (60%) had an overall higher frequency of mosaics versus siltstone (none), while siltstone (76.7%) had more gradients than quartzite (15%) when data were pooled across all days analysed (Table 2, Appendix Fig. A3).

**Table 2:** Frequencies of occurrence (%) for the three patterns of temperature difference on the lower surfaces of grey siltstone versus quartzite boulders (*n* = 20 per day sampled) for a subset of the total days sampled.

Rock type	Date	Maximum air temperature (°C)	Gradient	Temperature pattern (% of occurrence)	
				Mosaic	Limited heterogeneity
Grey siltstone	20/12/16	23	85	0	15
	28/1/17	29	85	0	15
	17/1/17	39	60	0	40
Quartzite	18/12/16	25	5	80	15
	29/1/17	30	15	70	15
	6/1/17	40	25	30	45

Overall occurrences of limited temperature heterogeneity were similar between grey siltstone (23.3%) and quartzite (25%) when data were pooled across all days analysed. Occurrences of limited heterogeneity were low (15%) for both rock types on the two cooler days sampled at each seashore, where gradients dominated on siltstone (85%) and mosaics on quartzite (≥70%, Table 2, Appendix Fig. A3). However, on the hottest day sampled for both rock types, occurrences of limited heterogeneity increased to 40–45% of all boulders sampled (Table 2), with a corresponding decrease in the occurrence of gradients and mosaics, especially mosaics on quartzite (Table 2, Appendix Fig. A3).

### Grey siltstone has a hotter maximum than quartzite

**Figure 4:**
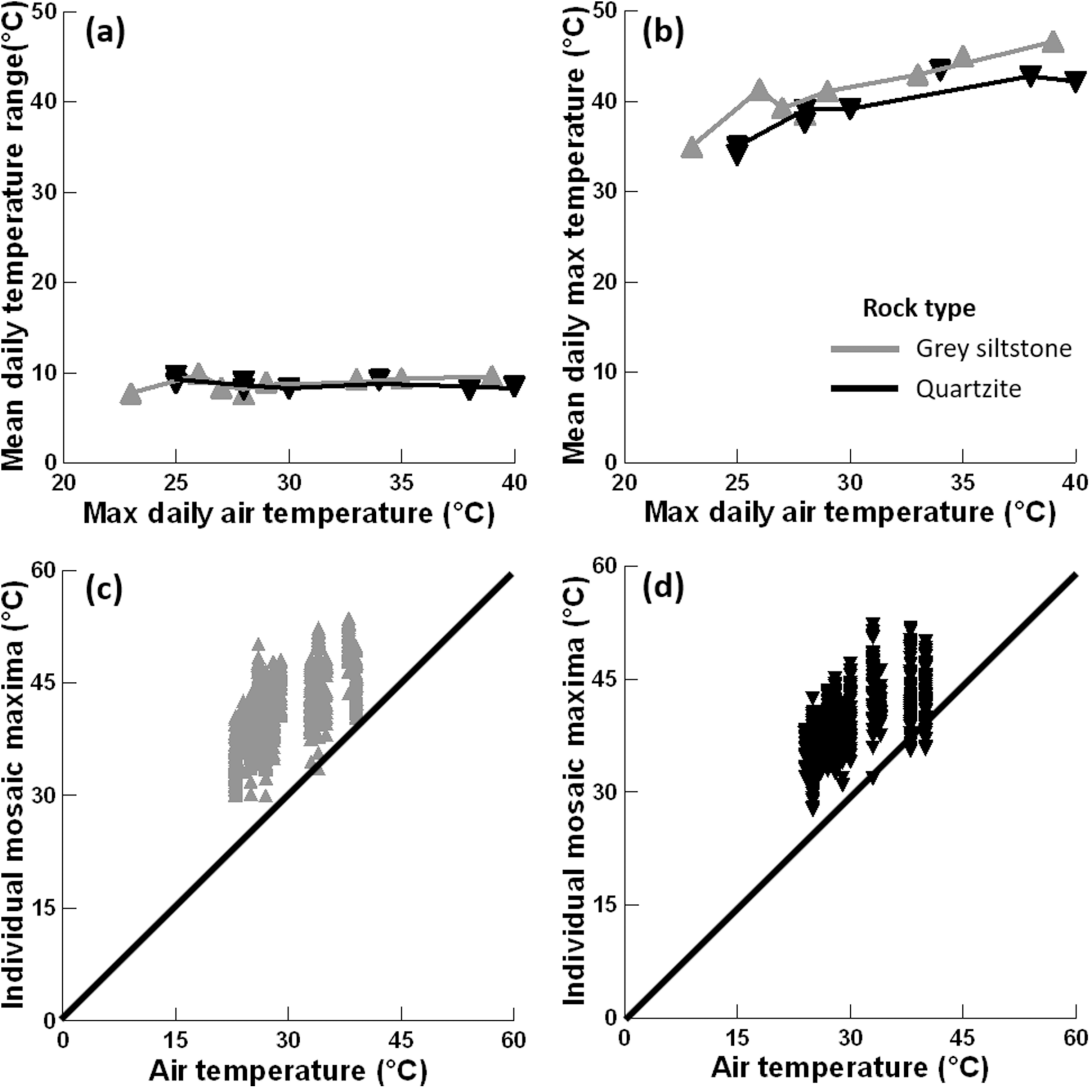
Relationships between substrate and air temperatures. (A) Mean temperature range on each day and maximum air temperature (LOWESS smoother fitted), (B) mean maximum temperature on each day and maximum air temperature (LOWESS smoother fitted), (C) maximum temperature for all replicate grey siltstone boulders and maximum air temperature (1:1 line shown), and (D) maximum temperature for all replicate quartzite boulders and maximum air temperature (1:1 line shown). Each y-axis extends to encompass the range of the raw data, with total *n* = 1152.

The mean temperature range on boulders across sampling days did not significantly differ between grey siltstone and quartzite (univariate PERMANCOVA *p*-value = 0.77, Figs. 3 & 4A, Appendix Table A1). Temperature range was not related to the daily maximum air temperature (PERMANCOVA covariate *p*-value = 0.64, Fig. 4A, Appendix Table A1). For grey siltstone, the smallest temperature range for an individual surface was 2.3 °C, while the largest temperature range was 18.5 °C. For quartzite, the smallest temperature range was 3.2 °C, while the largest temperature range was 16.9 °C.

Mean maximum temperature differed between rock types (PERMANCOVA *p*-value = 0.0073, Fig. 4B, Appendix Table A1), with grey siltstone (overall grand mean = 41.1 ± 0.2 °C) having significantly hotter maxima than quartzite (overall grand mean = 39.3 ± 0.2 °C). The mean maxima were significantly correlated with the maximum air temperatures on the day of sampling (PERMANCOVA covariate *p*-value = <0.0011, Appendix Table A1), with hotter maxima recorded on days with hotter air temperatures (Fig. 4B). The maximum temperature of each replicate boulder was generally hotter than the air temperature at sampling (Figs. 4C–4D). For grey siltstone, the coolest temperature measured was 20.1 °C, while the hottest temperature was 53.6 °C. For quartzite, the coolest temperature was 18.5 °C, while the hottest temperature was 52.2 °C.

### Target temperature differs from the maximum or average boulder temperature

**Figure 5:**
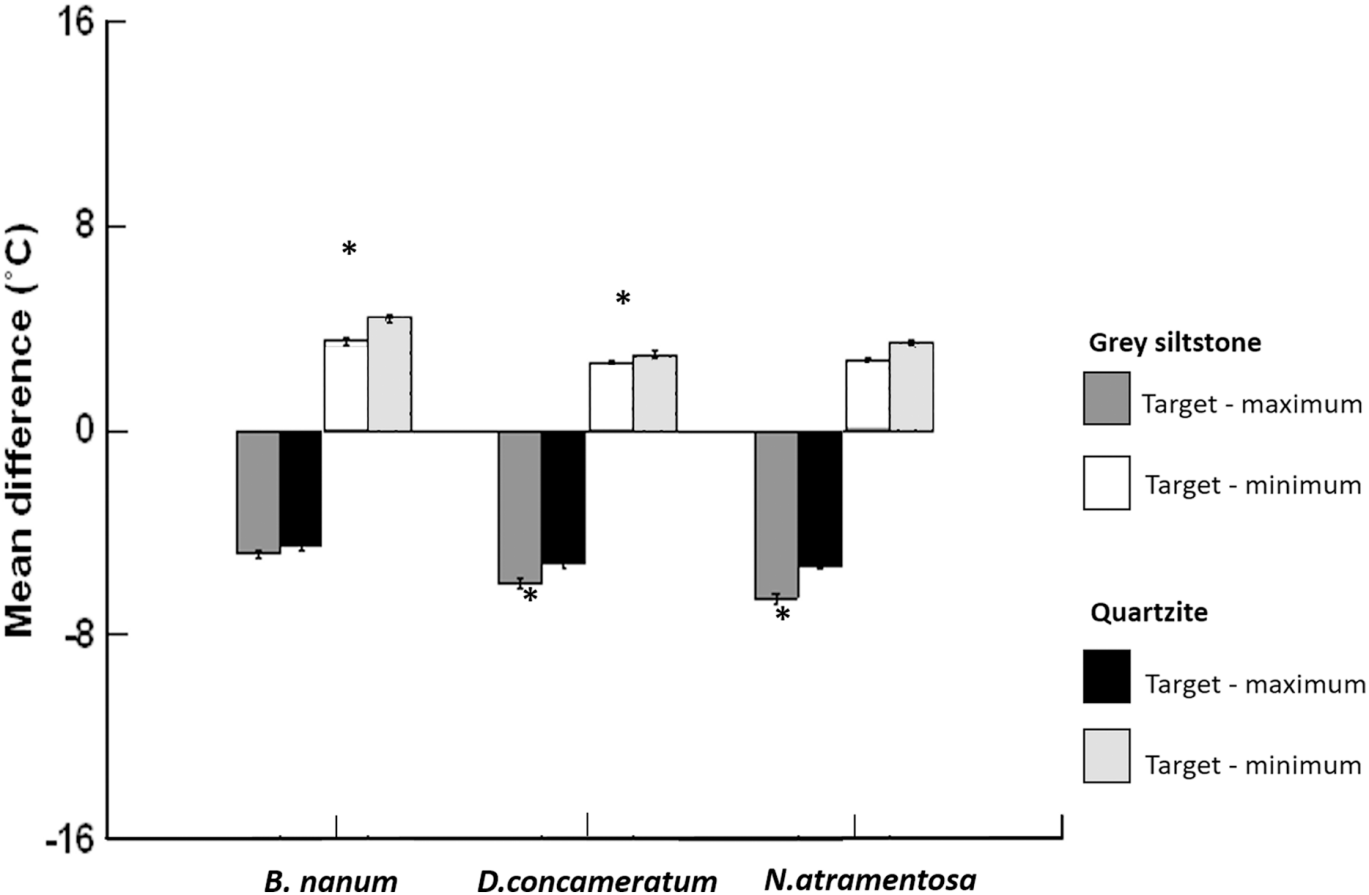
Grand mean ± SE (total *N* = 1389) for the differences between target temperature and maximum or minimum boulder temperatures on grey siltstone and quartzite for *Bembicium nanum*, *Diloma concameratum* and *Nerita atramentosa*. An asterisk (*) indicates significant difference detected between siltstone and quartzite for target—maximum or minimum temperatures for that snail species.

**Figure 6:**
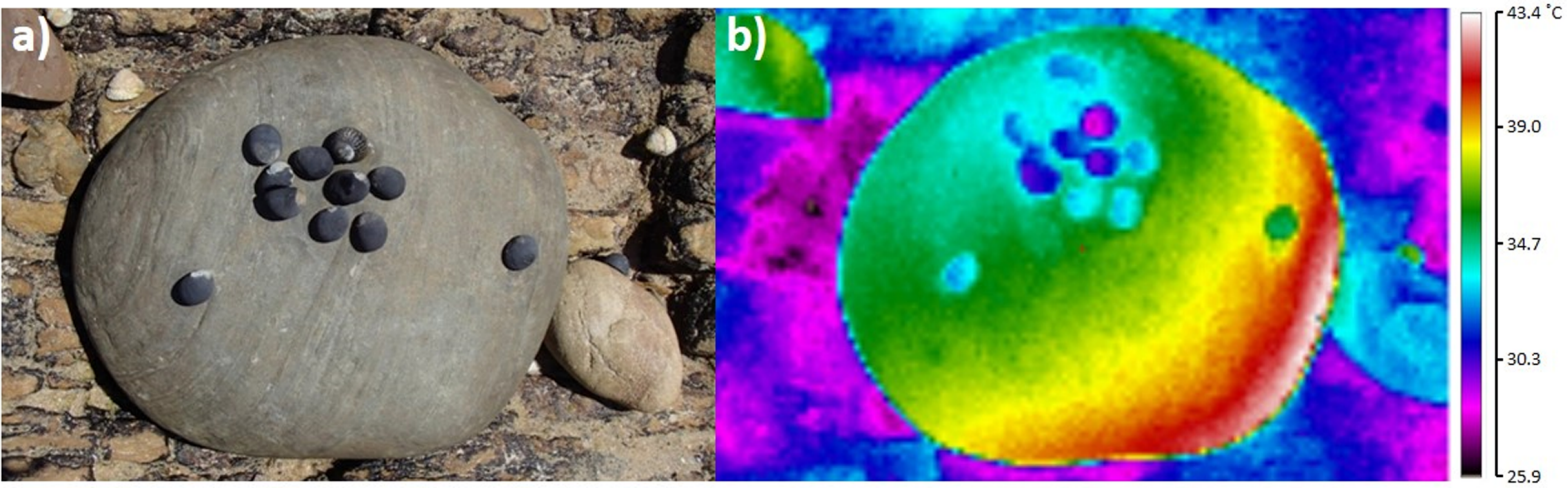
Example digital photograph (A) and thermal image (B) showing the spatial separation of snails from boulder-surface maxima for 10 *Nerita atramentosa* and one *Diloma concameratum* on the lower surface of grey siltstone. Ten out of eleven snails have occupied the cooler (shown by the blue and green colours) parts of this temperature gradient and are generally avoiding warmer (shown by the yellow and red colours) gradient locations.

Target temperatures, immediately adjacent to snails, were significantly cooler than the maximum temperature of boulders, on each day sampled for both rock types, for all three species (all paired *t*-test *p*-values = <0.001, Table 3, Figs. 5 & 6). Somewhat larger differences between target and maximum temperatures were recorded for areas of the rock surface occupied by *D. concameratum* and *N. atramentosa* than for *B. nanum* (Fig. 5, Table 1). For *B. nanum*, mean target temperatures were 4.1–5.7 °C and 4.0–4.8 °C cooler than mean maxima on each day sampled for grey siltstone and quartzite, respectively (Table 1). For *D. concameratum* mean target temperatures were 5.0–8.0 °C cooler on siltstone and 5.0–5.7 °C cooler on quartzite than mean maxima, while for *N. atramentosa*, mean target temperatures were 4.9–7.9 °C cooler on siltstone and 4.9–5.9 °C cooler on quartzite than mean maxima on each day sampled (Table 1). The hottest individual target temperatures measured immediately adjacent to snails for each species were 50.7 °C for *B. nanum*, 49.1 °C for *D. concameratum* and 46.3 °C for *N. atramentosa*. The association between target temperature and boulder maxima developed during the first hour that boulders were emersed, and was maintained during the four hour low-tide period. The position of snails on the boulders and associated target temperatures was not related to the amount of surface moisture retained on boulders (see Appendix for additional hypotheses tested and results).

**Table 3:** Paired *t*-tests testing for significant differences between target temperature and the maximum, minimum and average temperatures of boulders for *Bembicium nanum, Diloma concameratum* and *Nerita atramentosa* on grey siltstone and quartite.

Species:		*Bembicium nanum*						*Diloma concameratum*						*Nerita atramentosa*					
	Measure	Target—maximum		Target—minimum		Target—average		Target—maximum		Target—minimum		Target—average		Target—maximum		Target—minimum		Target—average	
Rock	Max air temp (°C)	${\rm{\bar{X}}}$	*p*-value	${\rm{\bar{X}}}$	*p*-value	${\rm{\bar{X}}}$	*p*-value	${\rm{\bar{X}}}$	*p*-value	${\rm{\bar{X}}}$	*p*-value	${\rm{\bar{X}}}$	*p*-value	${\rm{\bar{X}}}$	*p*-value	${\rm{\bar{X}}}$	*p*-value	${\rm{\bar{X}}}$	*p*-value
Grey siltstone	23	−4.09	**<0.001**	3.20	**<0.001**	0.17	0.586	−5.61	**<0.001**	2.77	**<0.001**	−0.89	**0.007**	−5.58	**<0.001**	2.35	**<0.001**	−1.06	**<0.001**
	26	−4.89	**<0.001**	3.76	**<0.001**	−0.22	0.540	−7.34	**<0.001**	2.93	**<0.001**	−1.62	**<0.001**	−7.19	**<0.001**	4.40	**<0.001**	−0.77	0.030
	27	−5.10	**<0.001**	3.49	**<0.001**	−0.13	0.728	−6.06	**<0.001**	2.05	**<0.001**	−1.30	**<0.001**	−6.23	**<0.001**	2.30	**<0.001**	−1.09	**<0.001**
	28	−4.26	**<0.001**	3.56	**<0.001**	0.20	0.489	−4.96	**<0.001**	2.61	**<0.001**	−0.48	0.037	−4.88	**<0.001**	2.46	**<0.001**	−0.53	0.067
	29	−5.66	**<0.001**	3.17	**<0.001**	−0.35	0.267	−6.46	**<0.001**	2.63	**<0.001**	−1.05	**<0.001**	−6.83	**<0.001**	2.14	**<0.001**	−1.29	**<0.001**
	33	−5.06	**<0.001**	3.82	**<0.001**	−0.22	0.543	−6.11	**<0.001**	2.72	**<0.001**	−1.10	**0.006**	−6.98	**<0.001**	2.82	**<0.001**	−1.60	**<0.001**
	35	−4.84	**<0.001**	4.25	**<0.001**	0.20	0.607	−7.22	**<0.001**	2.63	**<0.001**	−1.74	**<0.001**	−6.87	**<0.001**	2.61	**<0.001**	−1.37	**0.007**
	39	−4.67	**<0.001**	4.14	**<0.001**	−0.03	0.925	−8.01	**<0.001**	2.51	**<0.001**	−1.81	**0.001**	−7.91	**<0.001**	2.68	**<0.001**	−1.40	**<0.001**
Grand means (GM)		−4.82		3.67		−0.05		−6.36		2.59		−1.21		−6.56		2.72		−1.14	
Standard error of GM		0.14		0.15		0.12		0.19		0.10		0.11		0.17		0.11		0.11	
Quartzite	25	−4.65	**<0.001**	5.07	**<0.001**	0.63	0.045	−5.13	**<0.001**	4.58	**<0.001**	−0.26	0.244	−5.44	**<0.001**	4.72	**<0.001**	−0.34	0.221
	25	−4.36	**<0.001**	5.07	**<0.001**	0.88	**0.017**	−5.02	**<0.001**	3.61	**<0.001**	−0.48	0.064	−4.96	**<0.001**	3.62	**<0.001**	−0.47	0.067
	28	−3.96	**<0.001**	4.19	**<0.001**	0.53	0.114	−5.09	**<0.001**	3.15	**<0.001**	−0.79	**0.007**	−5.26	**<0.001**	3.19	**<0.001**	−0.79	**0.004**
	28	−4.64	**<0.001**	4.40	**<0.001**	0.26	0.299	−5.40	**<0.001**	3.70	**<0.001**	−0.55	**0.005**	−4.91	**<0.001**	3.93	**<0.001**	−0.49	0.072
	30	−4.68	**<0.001**	4.04	**<0.001**	0.16	0.600	−5.17	**<0.001**	2.92	**<0.001**	−0.77	**0.001**	−5.12	**<0.001**	3.13	**<0.001**	−0.87	**<0.001**
	34	−4.84	**<0.001**	4.97	**<0.001**	0.53	0.070	−5.71	**<0.001**	3.01	**<0.001**	−1.14	**<0.001**	−5.20	**<0.001**	3.59	**<0.001**	−0.77	**0.014**
	38	−4.41	**<0.001**	4.37	**<0.001**	0.51	0.113	−5.13	**<0.001**	2.33	**<0.001**	−1.06	**<0.001**	−5.89	**<0.001**	2.25	**<0.001**	−1.45	**<0.001**
	40	−4.29	**<0.001**	3.90	**<0.001**	0.18	0.442	−5.60	**<0.001**	2.68	**<0.001**	−1.15	**<0.001**	−5.52	**<0.001**	3.43	**<0.001**	−0.74	**0.010**
Grand means (GM)		−4.48		4.50		0.46		−5.26		3.28		−0.75		−5.29		3.48		−0.74	
Standard error of GM		0.12		0.14		0.10		0.13		0.11		0.08		0.12		0.11		0.09	

**Note:**

Significant differences (α = 0.025) between temperature measurements are shown in bold. ${\rm{\bar{X}}}$ = mean temperature difference.

For both *D. concameratum* and *N. atramentosa*, target temperatures were always cooler than average boulder temperatures on each day sampled for both rock types, with differences of up to 1.81 °C (Table 3). When observations are pooled across rock type, this difference was significant on 13 out of 16 days sampled for *D. concameratum* and 11 out of 16 days for *N. atramentosa*. In contrast, *B. nanum* target temperatures were generally similar (always <1 °C difference) to mean boulder temperatures on each day sampled for both rock types (Table 3). Target temperatures, adjacent to the snails, were significantly warmer than the minimum temperature of boulders, on each day sampled for each rock type, for all three species (paired *t*-test *p*-values = <0.001, Table 3, Fig. 5).

### Differences between target temperature and either thermal maxima or minima on boulders differed between grey siltstone and quartzite

Target temperatures were further from the maxima, nearer to the minima, and cooler relative to the average, on grey siltstone versus quartzite for all three snail species (Appendix Table A2). For *D. concameratum*, significantly larger negative mean differences were detected for grey siltstone than quartzite for target—maximum substrate temperatures (PERMANCOVA *p*-value = 0.0014, Appendix Table A1), while significantly smaller positive mean differences were detected for grey siltstone than quartzite for target—minimum temperatures (PERMANCOVA *p*-value = 0.0030, Fig. 5, Appendix Table A1). For *N. atramentosa*, significantly larger negative mean differences were identified for grey siltstone than quartzite for target—maximum temperatures (PERMANCOVA *p*-value = 0.0026, Appendix Table A1), although the smaller positive mean differences for siltstone than quartzite for target - minimum temperatures were not significantly different after Bonferroni correction (PERMANCOVA *p*-value = 0.0380, Fig. 5, Appendix Table A1). For *B. nanum*, this rock-related difference was not significant for mean target—maximum substrate temperatures (PERMANCOVA *p*-value = 0.12, Appendix Table A1), although significantly smaller positive mean differences were recorded on siltstone than quartzite for target—minimum substrate temperatures (PERMANCOVA *p*-value = 0.0019, Fig. 5, Appendix Table A1).

These mean differences between snail target temperature and the maxima and minima of boulders, for each rock type, were not strongly influenced by the air temperature at sampling for any species (smallest PERMANCOVA covariate *p*-value = 0.0329 for target—minimum temperatures for *D. concameratum*, which was not significant after Bonferroni correction, Appendix Table A1).

## Discussion

This research shows that lower boulder surfaces on the seashore often have heterogenous surface temperatures at a scale of millimetres to centimetres. On grey siltstone, gradients of surface temperature were most frequently observed, while on quartzite temperature mosaics were most common. Siltstone had hotter maximum surface temperatures than quartzite. At low tide, all three snail species retreated underneath boulders, with snail occupancy of under-boulder habitats associated with heterogenous under-boulder surface temperatures. All three species had target temperatures significantly cooler than the maxima, and similar to, or significantly cooler than the average temperature of boulders. These results provide clear evidence that snails generally avoided the hottest areas under boulders. Stronger associations between target and cooler boulder temperatures were recorded for *N. atramentosa* and *D. concameratum*, and on the hotter of the two rock types tested, grey siltstone.

The results from this field study corroborate the experimental findings described by Janetzki, Benkendorff & Fairweather (2021), in that lower boulder surfaces have heterogenous patterns of surface temperature, and that the maximum temperature underneath boulders is influenced by lithology. These results also complement a growing body of literature showing that substrate temperature on the seashore is heterogenous during periods of emersion (e.g. Garrity, 1984; Helmuth et al., 2006a; Denny et al., 2011; Marshall, Baharuddin & McQuaid, 2013). While the capacity of boulder lower surfaces to act as cooler refuge habitats has been recognised previously (Huey et al., 1989; Bertness, 1999; Chapperon & Seuront, 2011b), the temperature variability underneath them has only been described for terrestrial habitats (Huey et al., 1989), not for rocky seashores. Consequently, lower boulder surfaces also contribute to the heterogenous patterns of surface temperature observed on rocky seashores.

Intertidal gastropods under siltstone boulders were subjected to a slightly hotter range of temperatures than those under quartzite. Consequently, siltstone may offer a lower-quality thermal habitat on hot, sunny days than quartzite. This was reflected by snails on siltstone having stronger associations with boulder minima than on quartzite, suggesting that only locations further from the maxima and nearer to the minima offer an adequate thermal habitat under siltstone. The notion that different rock types provide different quality thermal habitats is supported by several intertidal studies, with Raimondi (1988) attributing the higher vertical seashore distribution of barnacles on granite compared to basalt to granite’s cooler temperatures. Moreover, the cooler body temperatures recorded by snail mimics on lighter-versus darker-coloured rocks was the result of overall cooler surface temperatures on lighter-coloured rocks (Marshall, McQuaid & Williams, 2010; Judge, Botton & Hamilton, 2011). Quartzite may also represent a better thermal habitat due to its temperature mosaics. Snails on siltstone would need to occupy cool positions at the end of a gradient to achieve maximum thermal relief (e.g. Fig. 3A). In contrast, as quartzite had small millimetre-to-centimetre scale patches of temperature across the entire surface (e.g. Fig. 3B), snails could potentially move much shorter distances to achieve thermal relief.

Establishing the thermal quality of under-boulder habitats is especially important given predictions of an increased frequency of heatwaves and hotter air temperatures associated with global climate change (IPCC, 2013). We have taken the first step by showing gastropod occupancy underneath boulders is associated with their heterogenous surface temperature. However, many important questions remain unanswered. Unlike previous studies, this study didn’t measure gastropod body temperature (e.g. Soto & Bozinovic, 1998; Caddy-Retalic, Benkendorff & Fairweather, 2011; Chapperon & Seuront, 2011a), so it remains unclear how heterogenous substrate temperatures underneath boulders directly affect gastropod body temperature. The thermal quality of each lower boulder surface may also be affected by subtle variations in other environmental parameters not measured here such as wave splash, relative humidity and wind speed/direction, which have been shown to affect both substrate and gastropod body temperature on rocky seashores elsewhere (Helmuth et al., 2006a; Coombes et al., 2013; Lathlean, Ayre & Minchinton, 2014). Moreover, this study specifically targeted lower boulder surfaces with gastropods attached to them. Consequently, this targeted sampling likely only investigated boulders with habitable thermal qualities, potentially excluding boulders deemed uninhabitable by gastropods because of their unfavourable thermal properties. Only after these knowledge gaps have been investigated will it be possible to make predictions regarding the benefits of heterogenous temperatures underneath boulders to the persistence of some intertidal biota under various climate change scenarios.

*Nerita atramentosa*, *D. concameratum* and *B. nanum* all retreated to the lower surfaces of boulders when emersed, with upper surfaces at low tide rarely occupied by any mobile biota (N. Janetzki, 2016, personal observation). This avoidance of dry, sun-exposed surfaces is consistent with observations made for a variety of gastropod species on seashores in Panama (Garrity, 1984) and Hong Kong (Yeung & Williams, 2012). It is thought that the protected lower surface of boulders, relative to more exposed substrates (e.g. upper boulder surfaces, rock platforms), afford intertidal gastropods their best chance of survival when emersed (Chapman, 2003; Chapperon & Seuront, 2011b). The substrate adjacent to all three snail species had target temperatures that were significantly cooler than the maxima, and that were similar to or cooler than the average temperature of boulders (see Fig. 5). This finding builds upon earlier studies investigating the interactions between intertidal gastropods and habitat temperature (e.g. Garrity, 1984; Jones & Boulding, 1999; Chapperon & Seuront, 2011b) by showing that behavioural thermoregulation in gastropods appears more complex than simply retreating to cooler refuge habitats. Instead, as previously reported for garter snakes under rocks in terrestrial habitats (Huey et al., 1989), there is a range of temperatures under intertidal boulders that mobile gastropods are associated with. Our results show that three snail species were associated with the cooler areas under boulders, clearly avoiding the hottest areas (e.g. Fig. 5).

This fine-scale association between snails and cooler locations has not previously been reported from under intertidal boulders. However, it is consistent with observations from other studies where periwinkles selected the coolest locations when offered centimetre-scale temperature gradients (Soto & Bozinovic, 1998; Jones & Boulding, 1999). As emersed *N. atramentosa* and *D. concameratum* aggregate in clumps to minimise desiccation and heat stress (Underwood, 1976; Chapperon, Le Bris & Seuront, 2013), the association found between snails and cooler substrate temperatures may be related to the aggregating behaviour of these species. As both species are inactive during low tide (Underwood, 1977), how they identify cooler locations within one hour of emersion (see Appendix), before boulders have reached their thermal maxima several hours later, remains unclear. Some congeners of these species will home to refuge habitats after feeding by following chemical cues in the mucus trails they create (Vannini & Chelazzi, 1978; Chelazzi, Deneubourg & Focardi, 1984; Chelazzi, Santina & Vannini, 1985). Therefore, after a period of high-tide feeding, the snails investigated here may home to specific locations under boulders using similar cues because of their favourable thermal properties.

Somewhat larger differences between target and maximum substrate temperatures were recorded for *N. atramentosa* and *D. concameratum* than for *B. nanum*. This may be related to the differing modes of behavioural thermoregulation employed by each species once they have retreated underneath boulders. In turn, this may affect each species thermal tolerance, and influence their interaction with the heterogenous temperatures underneath boulders. *N. atramentosa* and *D. concameratum* often retreat to cooler and damper habitats (Chapman, 2000; Chapperon & Seuront, 2011b; Chapperon, Le Bris & Seuront, 2013), where they may aggregate with conspecifics (Chapperon, Le Bris & Seuront, 2013) and evaporatively cool (McMahon, 1990). These behavioural responses to temperature suggest that *N. atramentosa* and *D. concameratum* require cooler refuge habitats, accounting for the larger differences that we recorded between target temperatures and maximum substrate temperatures for these species. In contrast, *B. nanum* may be able to withdraw into its shell and cement its aperture to the substratum with mucus (McMahon, 1990). Such a behavioural response suggests *B. nanum* can withstand hotter substrate temperatures, accounting for the smaller differences between target temperatures and maximum substrate temperatures recorded. It also helps to account for the higher-shore distribution of this species, as it can persist higher on the seashore where substratum temperatures are hotter.

Retreating under boulders and occupying cooler areas of temperature mosaics or gradients may not be sufficient to always avoid deleterious heat during daytime summer low tides, with some boulder and target temperatures on the hottest days sampled >50 °C in this study. Intraspecific variation in thermal tolerances and responses may enable some individuals to persist when exposed to these extreme substratum temperatures, with both Dong et al. (2017) and Moyen, Somero & Denny (2019) recording high intraspecific variation in thermal tolerances and responses in gastropod populations. Moreover, in conjunction with the behavioural thermoregulation by habitat selection observed here, each of these species may also employ other behavioural or physiological mechanisms to mitigate the risk of desiccation and heat stress. These include evaporative cooling or withdrawing deeply into the shell and sealing the aperture with the operculum (Vermeij, 1971; Suryanarayanan & Nair, 1979; McMahon, 1990). Consequently, these additional mechanisms may allow these snails to withstand exposure to short periods of extreme heat when emersed, such as those recorded under boulders in this study. Nevertheless, exposure to deleterious heat could be problematic, as some intertidal species presently live near their tolerable thermal limits (Helmuth et al., 2002; Somero, 2002; Madeira et al., 2012), and exceedance of these limits can result in issues ranging from reduced growth rates (Jones & Boulding, 1999; Lathlean, Ayre & Minchinton, 2013; Lamb, Leslie & Shinen, 2014) to mass mortality events (Harley, 2008; Seuront et al., 2019).

## Conclusions

This study shows that three snail species occupied cooler areas of temperature mosaics and gradients under boulders. Stronger associations between target temperatures adjacent to snails and boulder temperatures were recorded on grey siltstone than quartzite, and for the more-thermally sensitive *N. atramentosa* and *D. concameratum*. Therefore, associations between gastropods and substrate temperature also exist within the refuge habitats that gastropods retreat to, with these associations being both species and lithology specific. This new information allows us to better understand the associations between biota and heterogenous substrate temperature in the thermally-extreme and variable rocky intertidal zone, which is important given predictions of a warming climate associated with global climate change.

## Supplemental Information

10.7717/peerj.11675/supp-1 Supplemental Information 1 Raw data file for snail and boulder temperatures.

10.7717/peerj.11675/supp-2 Supplemental Information 2 Appendix.

## Additional Information and Declarations

### Field Study Permissions

The following information was supplied relating to field study approvals (i.e. approving body and any reference numbers): Field studies were approved by Primary Industries & Regions South Australia (PIRSA) under S115 Ministerial Exemption Number 9902638.

### Data Availability

The following information was supplied regarding data availability: Raw data are available in the Supplemental File.
