# Supplementary material for: Where three snail species attach while emersed in relation to heterogenous substrate temperatures underneath intertidal boulders
Source: PeerJ. 2021 Jul 9;9:e11675. doi: 10.7717/peerj.11675 (PMC8274491; doi:10.7717/peerj.11675)
Supplement: Supplemental Information 2 [file peerj-09-11675-s002.docx]

*The following appendix accompanies the article*

**Where three snail species attach while emersed in relation to heterogenous substrate temperatures underneath intertidal boulders**

**Nathan Janetzki*, Kirsten Benkendorff & Peter G. Fairweather**

*Corresponding author: [nathan.janetzki@outlook.com](mailto:nathan.janetzki@outlook.com)

*First additional hypothesis: any association between the target temperature and the maximum temperature does not change with time emersed.*

A consistent association between snails and maximum temperatures was recorded for each target species when sampling boulders emersed for at least four hours. However, this sampling was unable to identify when this association manifested during emersion, and whether it increased in strength with time emersed. Given these target species remain virtually motionless when emersed (Underwood 1977; McMahon 1990), we hypothesised that associations between snails and maximum temperatures would manifest shortly after boulders were emersed by the receding tide. To test this, some additional sampling using boulders emersed for one, two and four hours during a single low tide was completed. The same shore-parallel zones using the same boulder selection criteria described previously was used, with 30 independent lower surfaces, inhabited by each target species, sampled at each emersion time where possible (Table A2). To ensure the independence of measurements across emersion times, each flipped boulder was marked with chalk to ensure it was not re-sampled at a later time. As a larger number of inhabited surfaces per species had to be sampled (*N* = 90), only *N. atramentosa* and *B. nanum* were sampled, as *D. concameratum* was too scarce. Each target species was sampled on separate days, as it was not possible to find 30 surfaces inhabited by each target species within the one-hour sampling window between the one- and two-hour emersion times. Sampling was completed for grey siltstone only, with one day of cooler and hotter air temperatures sampled per species (Table A2).

To establish whether snail target or boulder temperatures changed across emersion times, separate PERMutational ANalyses Of VAriance (PERMANOVA) were completed for the untransformed measures of snail target temperature, boulder maxima, boulder temperature range, and for the difference between snail target – boulder maximum temperature for the factor Time emersed (i.e. 1, 2 and 4 hours; a fixed factor) on each day sampled. Euclidean distance resemblance matrices were prepared for each temperature variable, and permutations of residuals were completed using an unrestricted permutation of raw data model with 9999 permutations. When a significant difference was detected, pair-wise tests were used to distinguish significantly-different emersion times. Paired *t*-tests were used to test for a significant difference between snail target and maximum lower-surface temperature for each emersion time on each day sampled.

A range of temperatures was recorded on the lower surfaces of grey siltstone sampled after one, two and four hours of emersion (Figure A4). The size of this temperature range increased significantly with time emersed on all days except February 3, where a significant decrease with emersion time was observed (Figure A4a & Table A3, largest PERMANOVA *p*-value = 0.0025). Maximum temperature generally increased significantly with time emersed (Figure A4b & Table A3, all significant PERMANOVA *p*-values = 0.0001). As the temperature range increased and maxima warmed, there was a corresponding significant increase in *B. nanum* and *N. atramentosa* target temperatures at later emersion times (Figure A4c & Table A3, largest PERMANOVA *p*-value = 0.0003). For each emersion time on each day sampled, the association between snail target temperatures and boulder maxima was identical to that identified for H3, with *B. nanum* and *N. atramentosa* target temperatures significantly cooler than maxima (Figure A4d & Table A4, all paired *t*-test *p*-values = 0.000). The large differences between snail target and maximum substrate temperatures showed that snails avoided the hottest areas, with some evidence of this association strengthening with time emersed, especially for *N. atramentosa* (Figure A4d). There was a general trend for the difference between snail target and maximum surface temperatures to increase with emersion time, except on February 3 for *B. nanum*, where temperature differences decreased with emersion time (Figure A4d, Tables A3 & A4).

*Second additional hypothesis: any association between the target temperature and maxima does not change with surface condition (damp versus dry).*

As air temperatures became increasing hot, fewer boulders were inhabited by *N. atramentosa* or *D. concameratum*. Furthermore, on the surfaces that were inhabited, individual snails appeared to retreat to any damp patches that remained (NJ personal observation). Given these damp patches were also generally the coolest, we wondered whether *N. atramentosa* and *D. concameratum* were responding solely to substrate temperature, substrate moisture, or whether there was some sort of synergistic response. To test this, 30 naturally damp and 30 naturally dry surfaces inhabited by *N. atramentosa* were sampled on one day each of cooler and hotter air temperatures for grey siltstone (Table A2). Due to their scarcity on lower surfaces, especially on the hottest days sampled, it was not possible to also test this hypothesis for *D. concameratum*. Sampling was completed in the lower shore zone, using the same boulder-selection criteria detailed previously to select 30 damp and 30 dry surfaces, after boulders had been emersed for at least four hours during daytime low tides.

Paired *t*-tests were used to test for significant differences between snail target and boulder maximum temperatures for damp versus dry surfaces on each day sampled. This difference between target and maximum temperatures for replicate boulders was then used in univariate PERMANOVAs that tested whether this difference changed according to surface condition (damp versus dry; a fixed factor). PERMANOVAs were completed separately for each day sampled using Euclidean distance resemblance matrices derived from untransformed data.

Regardless of the surface condition (i.e. damp or dry), the association between *N. atramentosa* target temperature and the maximum temperature of grey siltstone was identical to that identified more-generally when testing H3 (Figure A5 & Table A4). All *N. atramentosa* target temperatures were significantly cooler than boulder maxima (Table A4). There was no evidence that this association between target temperature and boulder maxima differed between damp and dry surfaces, with the difference between snail target temperature and boulder maxima not differing between damp versus dry surfaces on either day sampled (PERMANOVA *p*-values = 0.18 on February 26 and 0.79 on February 27).

Table A1: Univariate PERMANCOVAs, where the maximum daily air temperature during low tide was included as a covariate (Air), testing for differences between Rocks for boulder temperature characteristics and the difference between snail target and boulder maximum or minimum temperatures for each snail species. Significant differences (α = 0.05 for boulder temperature characteristics and α = 0.025 for snail temperature differences) are shown in bold.

| Variable | Source | df | SS | MS | Pseudo-F | *p*-value |
| --- | --- | --- | --- | --- | --- | --- |
| Lower surface temperature range | Air | 1 | 0.12 | 0.12 | 0.22 | 0.6397 |
|  | Rock type | 1 | 0.05 | 0.05 | 0.09 | 0.7715 |
|  | Residual | 13 | 7.22 | 0.56 |  |  |
|  | Total | 15 | 7.40 |  |  |  |
|  |  |  |  |  |  |  |
| Lower surface maximum temperature | Air | 1 | 140 | 140 | 57.50 | **0.0001** |
|  | Rock type | 1 | 27 | 27 | 11.18 | **0.0073** |
|  | Residual | 13 | 32 | 2 |  |  |
|  | Total | 15 | 199 |  |  |  |
|  |  |  |  |  |  |  |
| *B. nanum* target – maximum temperature | Air | 1 | 0.01 | 0.01 | 0.05 | 0.8390 |
|  | Rock type | 1 | 0.49 | 0.49 | 2.82 | 0.1170 |
|  | Residual | 13 | 2.24 | 0.17 |  |  |
|  | Total | 15 | 2.74 |  |  |  |
|  |  |  |  |  |  |  |
| *B. nanum* target – minimum temperature | Air | 1 | 0.06 | 0.06 | 0.29 | 0.5908 |
|  | Rock type | 1 | 2.69 | 2.69 | 13.12 | **0.0019** |
|  | Residual | 13 | 2.66 | 0.20 |  |  |
|  | Total | 15 | 5.41 |  |  |  |
|  |  |  |  |  |  |  |
| *D. concameratum* target – maximum temperature | Air | 1 | 1.40 | 1.40 | 3.33 | 0.0888 |
|  | Rock type | 1 | 6.28 | 6.28 | 14.97 | **0.0014** |
|  | Residual | 13 | 5.46 | 0.42 |  |  |
|  | Total | 15 | 13.14 |  |  |  |
|  |  |  |  |  |  |  |
| *D. concameratum* target – minimum temperature | Air | 1 | 1.11 | 1.11 | 5.74 | 0.0329 |
|  | Rock type | 1 | 1.93 | 1.93 | 10.01 | **0.0030** |
|  | Residual | 13 | 2.51 | 0.19 |  |  |
|  | Total | 15 | 5.56 |  |  |  |
|  |  |  |  |  |  |  |
| *N. atramentosa* target – maximum temperature | Air | 1 | 1.68 | 1.68 | 4.55 | 0.0487 |
|  | Rock type | 1 | 7.18 | 7.18 | 19.50 | **0.0026** |
|  | Residual | 13 | 4.79 | 0.37 |  |  |
|  | Total | 15 | 13.65 |  |  |  |
|  |  |  |  |  |  |  |
| *N. atramentosa* target – minimum temperature | Air | 1 | 0.63 | 0.63 | 1.34 | 0.2791 |
|  | Rock type | 1 | 2.59 | 2.59 | 5.47 | 0.0380 |
|  | Residual | 13 | 6.16 | 0.47 |  |  |
|  | Total | 15 | 9.38 |  |  |  |

Table A2: The species, dates, air temperatures and number of snails (*n*) sampled on grey siltstone for hypotheses testing whether the associations between snail target temperature and boulder maxima changed with time emersed or surface condition. Surface condition for all the dashes were dry.

| Hypothesis tested | Species | Date | Time emersed (hours) | Surface condition | Air temperature at each emersion time (˚C) | *n* |
| --- | --- | --- | --- | --- | --- | --- |
| Time emersed | *Bembicium nanum* | 3/2/2017 | 1 | - | 26 | 30 |
|  |  |  | 2 | - | 26 | 30 |
|  |  |  | 4 | - | 24 | 30 |
|  |  | 28/2/2017 | 1 | - | 29 | 30 |
|  |  |  | 2 | - | 30 | 30 |
|  |  |  | 4 | - | 33 | 30 |
|  | *Nerita atramentosa* | 10/2/2017 | 1 | - | 27 | 25 |
|  |  |  | 2 | - | 29 | 26 |
|  |  |  | 4 | - | 32 | 30 |
|  |  | 14/2/2017 | 1 | - | 22 | 30 |
|  |  |  | 2 | - | 23 | 30 |
|  |  |  | 4 | - | 25 | 30 |
| Surface condition | *Nerita atramentosa* | 26/2/2017 | 4 | Damp | 22 | 30 |
|  |  |  | 4 | Dry | 22 | 30 |
|  |  | 27/2/2017 | 4 | Damp | 27 | 30 |
|  |  |  | 4 | Dry | 27 | 30 |

Table A3: Univariate PERMANOVAs testing for differences among emersion times (1, 2, and 4 hours) for target temperatures, the temperature range and maximum temperature of lower surfaces, and for the difference between target – boulder maximum temperature for each day sampled for each species on grey siltstone. When a significant difference was detected between emersion times, PERMANOVA pair-wise tests were used to identify which times were significantly different. Significant differences (α = 0.05) are shown in bold. The dashes denote where no pair-wise tests were completed, as no main effect for time emersed was detected.

| Species | Date | Hours compared | Target | Maximum | Range | Target - maximum |
| --- | --- | --- | --- | --- | --- | --- |
| *Bembicium nanum* | 3/2/2017 | 1,2,4 | **0.0003** | 0.0679 | **0.0010** | **0.0078** |
|  |  | 1,2 | **0.0044** | **-** | 0.2608 | **0.0379** |
|  |  | 1,4 | **0.0001** | **-** | **0.0003** | **0.0033** |
|  |  | 2,4 | 0.2750 | - | **0.0059** | 0.3885 |
|  |  |  |  |  |  |  |
|  | 28/2/2017 | 1,2,4 | **0.0001** | **0.0001** | **0.0014** | **0.0380** |
|  |  | 1,2 | **0.0001** | **0.0001** | **0.0073** | **0.0072** |
|  |  | 1,4 | **0.0001** | **0.0001** | **0.0011** | 0.0656 |
|  |  | 2,4 | **0.0001** | **0.0001** | 0.2722 | 0.5657 |
|  |  |  |  |  |  |  |
| *Nerita atramentosa* | 10/2/2017 | 1,2,4 | **0.0001** | **0.0001** | **0.0001** | **0.0001** |
|  |  | 1,2 | **0.0001** | **0.0001** | **0.0065** | **0.0429** |
|  |  | 1,4 | **0.0001** | **0.0001** | **0.0001** | **0.0001** |
|  |  | 2,4 | **0.0001** | **0.0001** | **0.0243** | **0.0136** |
|  |  |  |  |  |  |  |
|  | 14/2/2017 | 1,2,4 | **0.0001** | **0.0001** | **0.0025** | 0.0675 |
|  |  | 1,2 | **0.0001** | **0.0001** | 0.1475 | - |
|  |  | 1,4 | **0.0001** | **0.0001** | **0.0007** | **-** |
|  |  | 2,4 | **0.0001** | **0.0001** | **0.0304** | **-** |

Table A4: Paired *t*-tests testing for significant differences between target and maximum boulder temperature for *Bembicium nanum* and *Nerita atramentosa* on grey siltstone for time emersed or surface condition. Significant differences (α = 0.05) are shown in bold.  = mean temperature difference between target and maximum boulder temperature.

| Species | Date | Time emersed or surface condition |  | *p*-value |
| --- | --- | --- | --- | --- |
| *Bembicium nanum* | 3/2/2017 | 1 hour | -6.73 | **0.000** |
|  |  | 2 hours | -5.42 | **0.000** |
|  |  | 4 hours | -4.98 | **0.000** |
|  |  |  |  |  |
|  | 28/2/2017 | 1 hour | -5.17 | **0.000** |
|  |  | 2 hours | -6.51 | **0.000** |
|  |  | 4 hours | -6.19 | **0.000** |
|  |  |  |  |  |
| *Nerita atramentosa* | 10/2/2017 | 1 hour | -4.06 | **0.000** |
|  |  | 2 hours | -5.30 | **0.000** |
|  |  | 4 hours | -6.96 | **0.000** |
|  |  |  |  |  |
|  | 14/2/2017 | 1 hour | -4.37 | **0.000** |
|  |  | 2 hours | -4.98 | **0.000** |
|  |  | 4 hours | -5.56 | **0.000** |
|  |  |  |  |  |
|  | 26/2/2017 | Damp | -6.58 | **0.000** |
|  |  | Dry | -7.40 | **0.000** |
|  |  |  |  |  |
|  | 27/2/2017 | Damp | -6.31 | **0.000** |
|  |  | Dry | -6.16 | **0.000** |


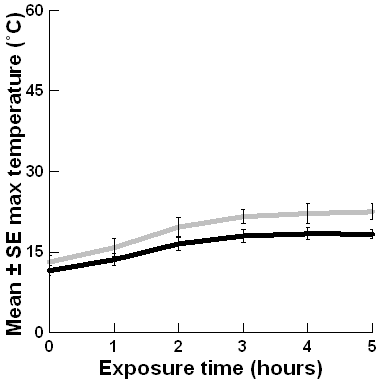


**2°C 5°C 8°C 9°C 12°C 13°C**

**a)**


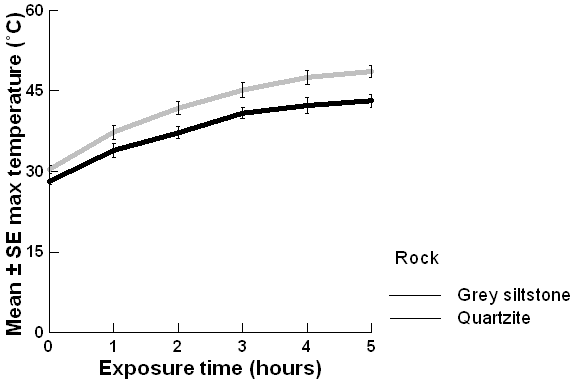


**33°C 36°C 38°C 40°C 40°C 39°C**

**b)**

**Rock type**

Grey siltstone

Quartzite

Figure A1: Mean ± SE maximum lower-surface temperatures for grey siltstone and quartzite boulders on sunny days with (a) cooler and (b) hotter air temperatures. Rock temperatures were measured at one hour intervals over five hours using thermal imagery in a common-garden experiment. The air temperature at the time of sampling is shown above each exposure time. Refer to Janetzki et al. (2021) for more details.


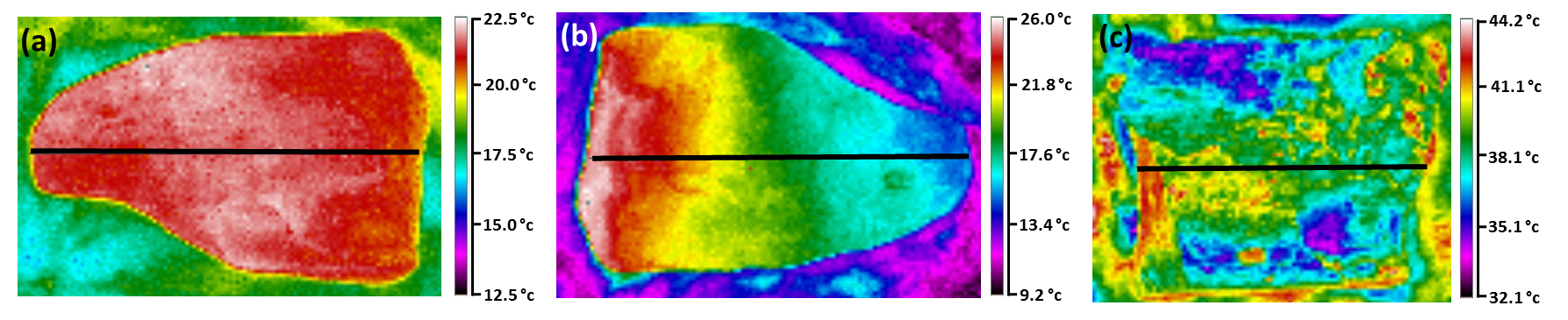


Figure A2: Thermal images showing qualitative patterns of temperature difference on boulder surfaces: (a) limited heterogeneity on the lower surface of grey siltstone; (b) temperature gradient on the lower surface of grey siltstone; and (c) temperature mosaic on the lower surface of quartzite. Temperature scales at right of each image are specific to each image. The black horizontal line denotes the transect drawn on each image to quantify temperature patterns. The sun is on the left of each image. Refer to Janetzki et al. (2021) for more details.


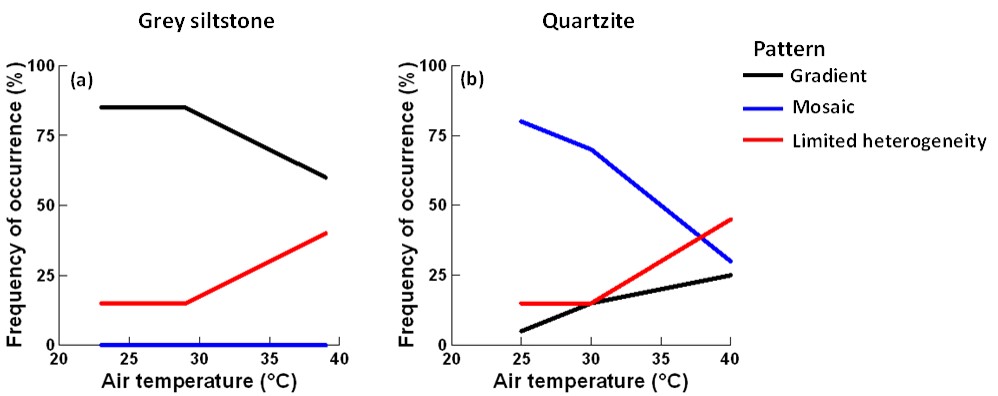


Figure A3: Frequencies of occurrence (%) for patterns of temperature difference on the lower surfaces of (a) grey siltstone and (b) quartzite boulders (*n* = 20 per day sampled) for three days of differing air temperature per rock type.

**3/2/2017**

**28/2/2017**

**10/2/2017**

**14/2/2017**

**Date**

*****

*****

*****

**3/2/2017**

**28/2/2017**

**10/2/2017**

**14/2/2017**

**Date**

***B. nanum***

***N.atramentosa***

***B. nanum***

***N.atramentosa***

**3/2/2017**

**28/2/2017**

**10/2/2017**

**14/2/2017**

**Date**

**3/2/2017**

**28/2/2017**

**10/2/2017**

**14/2/2017**

**Date**

*****

*****

*****

*****

*****

*****

*****

*****

*****

*****

*****

**(a)**

**(b)**

**(c)**

**(d)**


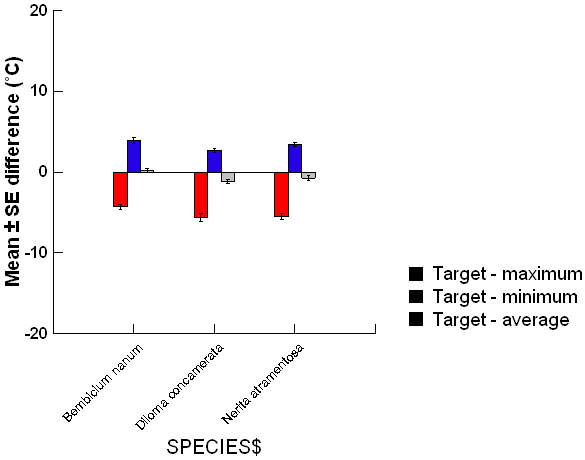


**1 hour**

**2 hours**

**4 hours**

**Time emersed**

Figure A4: The changes with time emersed on grey siltstone for the mean ± SE (a) lower-surface temperature range, (b) maximum lower-surface temperature, (c) target temperature and (d) difference between target – boulder maximum temperature. *Bembicium nanum* was sampled on February 3 & 28 while *Nerita atramentosa* was sampled on February 10 & 14. Each y-axis extends to encompass the range of the raw data. * = significant difference detected between emersion times.

**26/2/2017**

**27/2/2017**

**Date**

Figure A5: Mean ± SE difference for target – maximum temperature for damp versus dry lower surfaces of grey siltstone on each day sampled. All damp-dry comparisons were NS.
